# Bronchiectasis: an emerging global epidemic

**DOI:** 10.1186/s12890-018-0629-1

**Published:** 2018-05-22

**Authors:** Sanjay H. Chotirmall, James D. Chalmers

**Affiliations:** 1 Lee Kong Chian School of Medicine, Nanyang Technological University, 11 Mandalay road, Singapore, 308232 Singapore; 2 Division of Molecular and Clinical Medicine, School of Medicine, Ninewells Hospital and Medical School, Dundee, UK

**Keywords:** Bronchiectasis, Special issue, Infection, Non-tuberculous mycobacteria, Transplantation

## Abstract

Bronchiectasis has an increasing profile within respiratory medicine. This chronic and irreversible airways disease is common but suffers from a lack of evidenced based therapy for patients and, a lack of understanding of its inherent heterogeneity. Research focused on bronchiectasis must therefore be prioritized if we are to adequately address this evolving clinical problem. This special issue on bronchiectasis focuses on its clinical, microbiological and therapeutic aspects. By bringing together a unique collection of original research and review articles, we hope this issue will showcase international research efforts, encourage future research collaborations and stimulate debate. In doing so, we hope to bring greater attention to the urgent need for sustained investment into focused, dedicated and collaborative research platforms in bronchiectasis, an emerging “global epidemic”.

## Editorial

Bronchiectasis is experiencing a clinical and research renaissance. This emerging epidemic of chronic and progressive immune-infective-inflammatory airway destruction results in a vicious cycle of repeated exacerbations and irreversible damage that now clearly necessitates greater global focus and investment. The recent publication of the first international guidelines for its management is welcome, particularly as its prevalence and recognition continues to rise [1–3]. Over the forthcoming years, international health systems are likely to face great challenges in managing this group of patients and their associated costs. Therefore, it appears logical that more research, increased therapeutic trials and improved specialist services should be prioritised. Importantly, the clear need for greater bronchiectasis-focused research is further evidenced by the fact that no single licensed therapy can yet be recommended for patients. This is despite the large numbers of trialled agents necessitating re-consideration of our approach, research strategy, clinical focus and understanding of the disease’s inherent heterogeneity [4]. Significant knowledge gaps persist in key areas of disease including aetiology, pathogenesis and microbial infection. The rising profile of bronchiectasis is welcome. If we are to effect the true impact of this, we must continue to develop and curate large datasets across international populations to inform our clinical practice and importantly our research [5–7].

As academic respiratory research enters unchartered territory; one plagued by the challenge of overlap syndromes, the application of new “omics” technologies, improved tools to understand disease endo-phenotypes and a complex arena of bioinformatics, the relevance of these developing areas to bronchiectasis focused research must be considered [3, 8–10]. The quality and quantity of research in the field should increase in parallel to ensure a secure future for our patients and the care they receive. Airway infection remains a major challenge in bronchiectasis and emerging culture-independent sequencing technologies provide further insight into its complexity [5–7]. A deeper understanding of the functional implications of airway microbes in a lung affected by bronchiectasis is necessary and this goes beyond bacteria to include non-tuberculous mycobacterial species, viruses and even fungi [11–14].

To add to the growing focus and interest in bronchiectasis, we dedicate this special issue to further promote its resurgence. This issue includes six original and three commissioned review articles organized into three thematic areas of importance: (1) clinical disease (2) airway infection and (3) lung transplantation.

### Clinical disease

In this special issue, the clinical aspects of bronchiectasis are covered from both the physician and patient perspective. The authoritative review by Schäfer *and colleagues* reviews bronchiectasis pathogenesis, its imaging and clinical characteristics from both a cystic fibrosis (CF) and non-CF perspective across a diverse range of aetiologies [15]. In a patient focused article, Hester et al. address the issue of adherence to the multi-modal treatment options used in the care of bronchiectasis patients by reviewing the provision of and requirement for patient education [16]. Minimal work to date has been performed in bronchiectasis focused in this area especially when compared to other chronic respiratory conditions such as asthma and chronic obstructive pulmonary disease (COPD). The information deficits uncovered in this qualitative approach clearly represent one of the key barriers to the implementation of bronchiectasis self-management. Further original qualitative work is provided by Dudgeon et al. whose study focused on the patient’s perspective of health-related quality of life (QOL) [17]. Semi-structured interviews elucidated that bronchiectasis-related symptoms are highly individual and variable, a feature that current available treatment and QOL tools do not adequately capture.

### Airway infection

Infection is an established driver of exacerbations and disease progression in bronchiectasis, however, while acknowledged, geographic variation in disease has not been previously reviewed. Chandrasekaran et al. performed a comprehensive review of this understudied area of disease heterogeneity that has emerged in recent times, further evidenced by the contrasting results from the RESPIRE 1 and 2 trials performed across geographically distinct regions [18–21]. Three original articles focused on non-tuberculous mycobacteria (NTM), viruses and paediatric microbiomes are further important additions to this special issue. Lim *and colleagues* illustrate that NTM profiles in an Asian setting (Singapore) are unique with *M. abscessus* commonest and associated with high rates of pulmonary tuberculosis, itself a major contributor to the bronchiectasis burden in Asia [22]. Mitchell et al. examined viruses in stable and exacerbating bronchiectasis in a pilot study [23]. The strength of this work is a weakness of prior studies in this area: an assessment of viral prevalence in the stable clinical state. The authors detect viruses at high frequency from respiratory secretions and exhaled breath, even in the stable state and with the absence of clinical symptoms, questioning their ‘true’ relevance in bronchiectasis. Longitudinal studies are clearly required including those focused on the host ‘virome’ to provide greater clarity to these findings. In contrast to the ‘virome’ , the microbiome in bronchiectasis has been better studied and Masekela et al. further add to this by assessing the lung microbiome in children with human immunodeficiency virus (HIV)-related bronchiectasis, a field where minimal data currently exists [24]. This study showed a less diverse and largely heterogeneous microbiome dominated by Proteobacteria when compared to a small control group with CF illustrating the contrasting pathologies leading to bronchiectasis in the context of susceptibility to infection.

### Lung transplantation

Lung transplantation is a viable therapeutic option in bronchiectasis and only a few publications have reported on this management strategy for end-stage disease. This issue includes two original articles, one assessing transplant outcomes and the other international practice. Birch et al. report the outcomes of lung transplantation over a period of more than two decades from a major European transplant referral centre [25]. The data show that lung transplantation for bronchiectasis is useful, associated with good lung function and importantly survival, which is comparable to other bilateral lung transplant recipients suffering from other causes of end-stage respiratory failure. Tissot *and colleagues* focused on the difficult issue of NTM infection in CF patients suitable for transplant listing and ascertaining current international practice [26]. NTM infection is complex, therapeutically challenging and associated with poorer disease outcome in CF however the suitability of such patients for lung transplantation is poorly characterized. Data in this issue illustrate that across international transplant centres a lack of standards exists, leading to variable practices. Therefore, an urgent need for better quality data to aid clinical decision making exists to inform equitable access to lung transplant for NTM-infected CF patients.

The once silent bronchiectasis epidemic continues to gain momentum. Its recent global resurgence necessitates dedicated research and sustained investment if we are to improve the clinical care delivered and our understanding of its pathogenesis. This special issue dedicated to bronchiectasis aims to reaffirm its growing profile through the varied collection of articles focusing on its clinical, microbiological and therapeutic aspects. The time has come to control the evolving bronchiectasis epidemic, and to do so requires a collective manifesto of using evidenced based research to deliver safe and effective therapy to suffering patients with this life-threatening disease.

## Abbreviations

CF: Cystic fibrosis
COPD: Chronic obstructive pulmonary disease
HIV: Human immunodeficiency virus
NTM: Non-tuberculous mycobacteria
QOL: Quality of life
